# High Indoleamine 2,3-Dioxygenase Is Correlated With Microvessel Density and Worse Prognosis in Breast Cancer

**DOI:** 10.3389/fimmu.2018.00724

**Published:** 2018-04-17

**Authors:** Lijuan Wei, Shanshan Zhu, Menghui Li, Fangxuan Li, Feng Wei, Juntian Liu, Xiubao Ren

**Affiliations:** ^1^Department of Cancer Prevention Center, Tianjin Medical University Cancer Institute and Hospital, Tianjin, China; ^2^National Clinical Research Center for Cancer, Tianjin, China; ^3^Key Laboratory of Cancer Prevention and Therapy, Tianjin, China; ^4^Tianjin Clinical Research Center for Cancer, Tianjin, China; ^5^Hexian Memorial Hospital of Panyu District, Guangzhou, China; ^6^Department of Immunology, Tianjin Medical University Cancer Institute and Hospital, Tianjin, China; ^7^Department of Biotherapy, Tianjin Medical University Cancer Institute and Hospital, Tianjin, China

**Keywords:** breast cancer, indoleamine 2,3-dioxygenase, microvessel density, CD105, human umbilical vein endothelial cells

## Abstract

Indoleamine 2,3-dioxygenase (IDO), which catalyzes the breakdown of the essential amino acid tryptophan into kynurenine, is understood to have a key role in cancer immunotherapy. IDO has also received more attention because of its non-immune functions including regulating angiogenesis. The purpose of this study was to investigate the effects of IDO on microvessel density (MVD), and to explore its prognostic role in breast cancer. We showed IDO expression was positively correlated with MVD labeled by CD105 (MVD-CD105) rather than MVD labeled by CD31 (MVD-CD31) in breast cancer specimens. Both IDO expression and MVD-CD105 level were associated with initial TNM stage, histological grade, and tumor-draining lymph nodes (TDLNs) metastasis in breast cancer. In the prognostic analysis, TDLNs metastasis, an advanced TNM stage (III) and high histological grade (III) significantly predicted shorter survival in univariate analysis. Concentrating on IDO and MVD, the patients with IDO expression or high MVD level had poorer prognosis compared with no IDO expression [*P* = 0.047 for progress-free survival (PFS)] and low MVD level (*P* = 0.019 for OS); the patients with IDO expression and high MVD level had a tendency with shorter overall survival when compared with non IDO expression, low MVD level, or both (*P* = 0.062 for OS). In multivariate analysis, an advanced TNM stage (III) was significantly associated with shorter 5-year survival rate of PFS (HR: 0.126, 95% CI: 0.024–0.669, *P* = 0.015). In order to verify the phenomenon of IDO promoting angiogenesis, we contained the study *in vitro*. We detected the expression of IDO mRNA in breast cancer cell lines and measured the concentration of tryptophan and kynurenine in the supernatants of MCF-7 by high performance liquid chromatography. The ratio of Kyn and trp (kyn/trp) was calculated to estimate IDO-enzyme activity. MCF-7 cells, which produce high level of IDO and metabolize tryptophan, promoted human umbilical vein endothelial cells (HUVEC) proliferation significantly in co-culture system. Meanwhile IDO could upregulate the expression of CD105 in HUVEC, which was downregulated after adding IDO inhibitor, 1-methyl-d-trytophan. These results suggest that IDO could promote angiogenesis in breast cancer, providing a novel, potentially effective molecular or gene therapy target for angiogenesis inhibition in the future.

## Introduction

Indoleamine 2,3-dioxygenase (IDO), which catalyzes the breakdown of the essential amino acid tryptophan into kynurenine, is understood to have a key role in cancer immunotherapy because of its role in enabling cancers to evade the immune system. As we know, a variety of human tumors, including breast cancer, overexpress IDO, both in tumor cells or in tumor-associated cells (e.g., DCs, stromal cells, endothelial cells). IDO can suppress effector T cells by depleting tryptophan and producing tryptophan metabolites, and IDO can effect systemic tolerance by activating circulating Treg cells. Most studies have shown that high IDO expression in tumor tissues usually correlates with a significantly worse prognosis in patients (1, 2). Thus, blocking or ablating the IDO immune-inhibitory pathway may be an important anticancer immunotherapy strategy, and several IDO inhibitors are currently in clinical trials (3).

Tumor angiogenesis is an important factor for tumor growth and metastasis, and angiogenesis has been strongly linked with worse prognosis for many years (4). Neoangiogenesis in malignancies reflects the capacity of tumors to produce blood-borne metastases. Microvessel density (MVD) has been associated with worse prognosis in different cancers, such as colorectal cancer (5), endometrial cancer (6), and esophageal carcinoma (7). But, a clear marker for angiogenesis has yet to be identified. CD31 is a transmembrane glycoprotein, a member of the immunoglobulin superfamily, and is expressed on early and mature vascular endothelial cells (8). CD105 is a homodimeric transmembrane glycoprotein, which belongs to the zona pellucida family of extracellular proteins, is expressed in activated endothelial cells in culture and in tumor microvessels (9). Di Paolo et al. observed a significant positive correlation between the MVD individually measured by CD31 and CD105 in pediatric rhabdomyosarcoma (10). But in Mohamed et al.’s study, the roles of CD105 and CD31 in the prognosis of colorectal cancer cases were different. There was a statistically significant association between recurrence rates with CD105 but not with CD31 (11). It is still unclear which is a better marker for tumor progression and prognosis, CD31 or CD105.

De Palma et al. reported that the tumor microenvironment may promote tumor angiogenesis (12). IDO has received recent attention because it has non-immune functions including regulating angiogenesis. The first report was in 1978 by Li et al. who reported that in the co-culture system of vascular endothelial cells and fibroblasts, forced IDO expression promoted the luminal formation of vascular endothelial cells through tryptophan depletion (13). Nonaka et al. (14) established an IDO-expressing cell to examine, both *in vitro* and *in vivo*, relationships between IDO and tumor cell growth. Tumor cells expressing IDO showed greater formation of new blood vessels than control tumors. This indicates that, in addition to its effects on immunotolerance, IDO may also promote tumor progression through angiogenesis. Su et al. found that IDO could promote the attachment, migration, invasion and vasculogenic mimicry (VM) formation of 2LL cells *in vitro*. They explored that the mechanism of IDO promoting angiogenesis is associated with the JAK2/STAT3 pathway and its downstream genes MMP2/MMP-9 (15). So far, only a few studies have explored the relationship between IDO and angiogenesis in tumors, but we are unaware of any findings in the context of breast cancer.

The aims of the present retrospective study were to examine the relationship between IDO and the microvascular density and to evaluate its clinical significance and prognostic value in breast cancer patients. Furthermore, we explored the effect of IDO on human umbilical vein endothelial cells (HUVEC). More specifically, we aimed to explore whether IDO expression will promote tumor progression through neoangiogenesis induction which was represented by the microvascular density assessed by CD105 staining.

## Materials and Methods

### Patients and Tissues

A total of 65 paraffin sections were collected from breast cancer patients aged 30–79 years (median age, 52 years) who underwent surgery in the Tianjin Medical University Cancer Institute and Hospital from 2012 to 2013. These patients did not receive chemotherapy or hormone therapy before surgery. The study was approved by the Ethics Committee of Tianjin Cancer Institute and Hospital. Each patient gave written informed consent. Surgical pathology reports were reviewed according to the 6th edition of the AJCC Cancer Staging Manual. According to WHO 2012 tumor classification criteria, most tumors were classified as ductal carcinoma (76.9%). The median follow-up times for survival were 68 months (range: 20–97 months).

### Immunohistochemistry (IHC) for IDO, CD31, and CD105 Expression

Formaldehyde-fixed, paraffin-embedded tissue samples were sectioned into 4 µm slices and affixed on glass slides. Immunohistochemical staining was performed according to instruction manuals. After being heated for half an hour at 56°C, the slides were deparaffinized in xylene and rehydrated through graded alcohol. Antigens were retrieved by heating in citrate buffer for 20 min and endogenous peroxidase activity was quenched in a bath of methanol and hydrogen peroxide for 30 min. The slides were incubated with mouse anti-human IDO monoclonal antibody (Chemicon Corporation, MA, USA) at concentration of 1:300 or mouse anti-human CD31 monoclonal antibody (DAKO, Glostrup, Denmark) at concentration of 1:200 or rabbit anti-human CD105 polyclonal antibody (Abcam, UK) at concentration of 1:200 at 4°C overnight. The antibodies were detected by a biotinylated secondary antibody labeled with streptavidin-horseradish peroxidase, a DAB staining kit was used for the visualization of immunoreactive cells. The primary antibody was substituted with PBS for the negative control.

### Evaluation of IDO and MVD

For semi-quantitative analysis, staining rate (SR) and staining index (SI) both were indicators to describe protein expression of IDO. SRs referred to the percentages of positive samples in all samples. IDO expression score was separated from 0 to 3 (0 = <5% of tumor cells were stained; 1 = 5–30% were stained; 2 = 30–70% were stained; 3 = >70% were stained). SI was determined upon the average of at least 5 high-powered fields (400× magnification) and separated from 0 to 3 (0: no staining; 1: mild staining; 2: moderate staining; 3: strong staining). Finally, a score was calculated as the sum of SR and SI, with ≤1 defined as negative expression and >2 defined as positive expression.

Microvessel count was assessed in five high-powered fields (400× magnification) according to Weidner et al. (16) method. The mean value of the vessel count in the five fields was retained as the final value. Luminal diameter greater than eight erythrocytes or wall of the smooth muscle surrounded by large vessels are not included in the counting range.

An Olympus BX51 microscope was used for image acquisition and data analysis. All samples were evaluated by two experienced pathologists independently to ensure the reproducibility of the IHC analysis.

### Cell Culture

All the cancer cell lines, MDA-MB-231, MDA-MB-435S, MDA-MB-453, SK-Br-3, ZR-75-1, T47D, and MCF-7, were purchased from China National Infrastructure of Cell Line Resource. MDA-MB-231, MDA-MB-435S and MDA-MB-453 were cultivated in L15 medium supplemented with Glutamine, SK-Br-3 was cultivated in DMEM, and ZR-75-1, T47D, and MCF-7 were cultivated in RPMI 1640. All these mediums (Gibco, Invitrogen Corp., Carlsbad, CA, USA) contained 10% fetal bovine serum (Hyclone, Logan, UT, USa). Culture conditions were 37°C and 5% CO_2_.

### IDO mRNA Assay

The mRNA expression of IDO gene in breast cancer cell lines was analyzed using RT-PCR. Trizol Reagent (Invitrogen, Carlsbad, CA, USA) was used to extract total RNA, and MMLV reverse transcriptase (Promega, Madison, WI, USA) was used to reverse transcribe to cDNA. Expression levels of target genes were quantified using the SYBR Premix Ex Taq system (Takara Bio, Tokyo, Japan) following the manufacturer’s instructions. The primers applied in this assay were: IDO (188 bp), sense 5′-CATCTGCAAATCGTGACTAAG-3′; antisense 5′-CAGTCGACACATTAACCTTCCTTC-3′. β-actin (186 bp) was used as an internal control; sense 5′-TGGCACCCAGCACAATGAA-3′; antisense 5′-CTAAGTCATAGTCCGCCTAGAAGCA-3′. The specificity of the primers was verified and described in our prior papers (17, 18). The thermal cycling program was listed below: initial denaturalization at 94°C for 5 min, then 94°C for 30 s, 58°C for 30 s, and 72°C for 45 s for 35 cycles; after the last cycle, 72°C for 10 min. All tests were repeated at least three times.

### IDO Activity Assay

The concentration of tryptophan and kynurenine in the supernatants of MCF-7 or no cells culture were detected by high performance liquid chromatography (HPLC) on an Agilent HP1100 (Agilent Technologies, Palo Alto, CA, USA) series instrument using reversed phase C18 columns with a UV detector (225 nm). Retention times for kyn and try were 8.65 and 16.51 min, respectively. The ratio of Kyn and trp (kyn/trp) was calculated to estimate IDO-enzyme activity.

### HUVEC Cell Proliferation and CD105 Expression in Co-Culture System

To ensure stable growth characteristics, tumor cells were seeded into the upper layer of a transwell plate at a density of 0.85 × 10^4^/cm^2^ in 0.5 ml of culture medium while HUVEC were seeded into the lower layer at a density of 3.5 × 10^4^/cm^2^ in 1.5 ml of culture medium. Cells were cultured in RPMI 1640 medium containing 10% fetal calf serum. Starting after seeding, HUVEC were harvested using 0.05% trypsin-EDTA every 24 h, and counted with a hemocytometer to draw a growth curve. Flow cytometry was used to detect the expression of CD105 on the surface of HUVEC at different times. After co-culturing for 24, 48, and 72 h, HUVEC were labeled using following FITC-conjugated mouse anti-human antibodies in fluorescence activated cell sorter buffer (BD Biosciences, USA) for 30 min at room temperature; the antibody was anti-CD105 (Clone 266). FITC-conjugated and purified anti-mouse IgG1 (BD Biosciences, USA) were used as an isotype control. HUVEC were analyzed using FACS Calibur and FACS CantoII instruments (BD Biosciences, USA) and FlowJo software was used to determine the percentage of positive cells.

### Statistical Analyses

The statistical analysis was performed using the SPSS version 17.0 (IBM Corp., Armonk, NY, USA) software. Chi-square and Fisher’s exact tests were used to compare groups on categorical variables. Independent samples *t*-tests were used to compare groups on continuous variables. Spearman’s rank-order tests and liner regression analyses was used to assess correlations between continuous variables, while Pearson’s test was used to assess correlations between continuous variables and classification of variables. Survival analysis was performed using Kaplan–Meier analyses with Log-rank test. Multivariate analysis was performed with Cox’s regression. All statistics were twosided and statistical significance was defined as *P* < 0.05.

## Results

### IDO, CD31, and CD105 Expression in Breast Cancer Tissue

**Figure 1:**
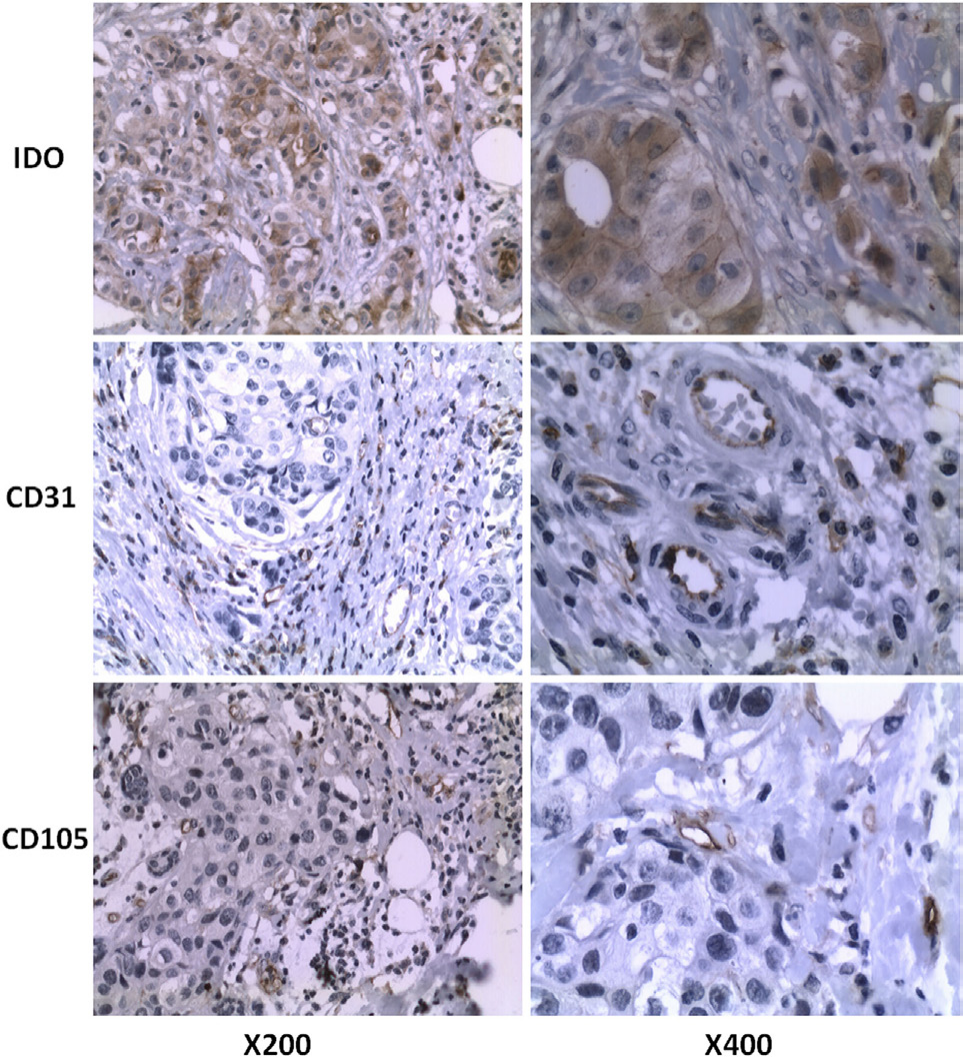
IDO, CD31, and CD105 protein expression of breast cancer tissues were assessed by immunohistochemistry. Formalin-fixed and paraffin-embedded sections were stained using the murine monoclonal antibody against human IDO. Original magnification, ×200 and ×400, respectively.

Figure 1 shows the expression status of IDO, CD31, and CD105 in human breast cancer paraffin tissues. IDO protein expression was detected in cytoplasm and mainly confined to tumor cells and occasional tumor-infiltrating lymphocytes, but not in normal adjacent tissues (Figure S1 in Supplementary Material). Most (64.6%, 42/65) of the breast cancer specimens were positive for IDO staining. The anti-CD31 and anti-CD105 antibody highlighted the microvessels by staining endothelial cell membrane. Clusters of endothelial cells stained with CD105 or CD31 that were clearly separated from adjacent microvessel were considered microvessels. CD105 was distributed regularly along the small vessels as thin and linear deposits, while CD31 was mainly expressed in larger vessels.

### Association Between IDO and MVD

**Figure 2:**
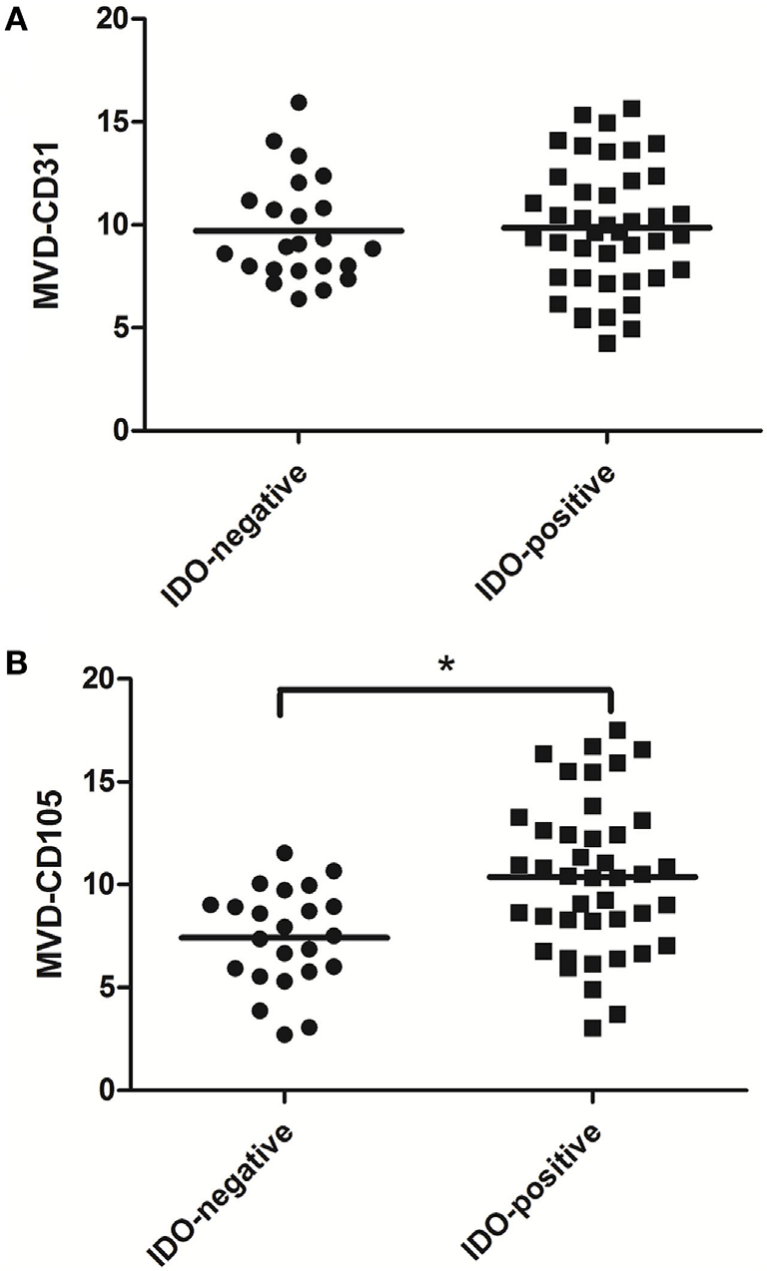
Scatter plot of microvessel density (MVD)-CD31 level and MVD-CD105 level in breast cancer tissue with indoleamine 2,3-dioxygenase (IDO) negative and positive expression. The MVD-CD105 level was higher in tissue with IDO positive than IDO negative **(B)**, whereas MVD-CD31 did not differ in IDO-expressing and non-expressing tissues **(A)**.

The association between IDO expression and MVD measured by CD31 or CD105 (MVD-CD31 or MVD-CD105, respectively) in tumor microenvironment were shown in Table 1. The MVD-CD105 level was higher in tissue with IDO positive than IDO negative (*P* < 0.001), whereas MVD-CD31 did not differ in IDO expression and non-expression tissues (Figure 2).

**Table 1:** Indoleamine 2,3-dioxygenase (IDO) expression associated with microvessel density (MVD) level in tumor microenvironment.

IDO expression	*N*	MVD-CD31	MVD-CD105
Negative	23	9.72 ± 3.52	7.46 ± 2.17
Positive	42	9.84 ± 3.52	10.90 ± 2.74
*t*		0.096	5.110
*P*		0.920	<0.001

*The mean value of the vessel count in the five high-powered fields (400× magnification) was retained as the final MVD value*.

### Associations of IDO and MVD-CD105 With Clinicopathologic Characteristics

**Figure 3:**
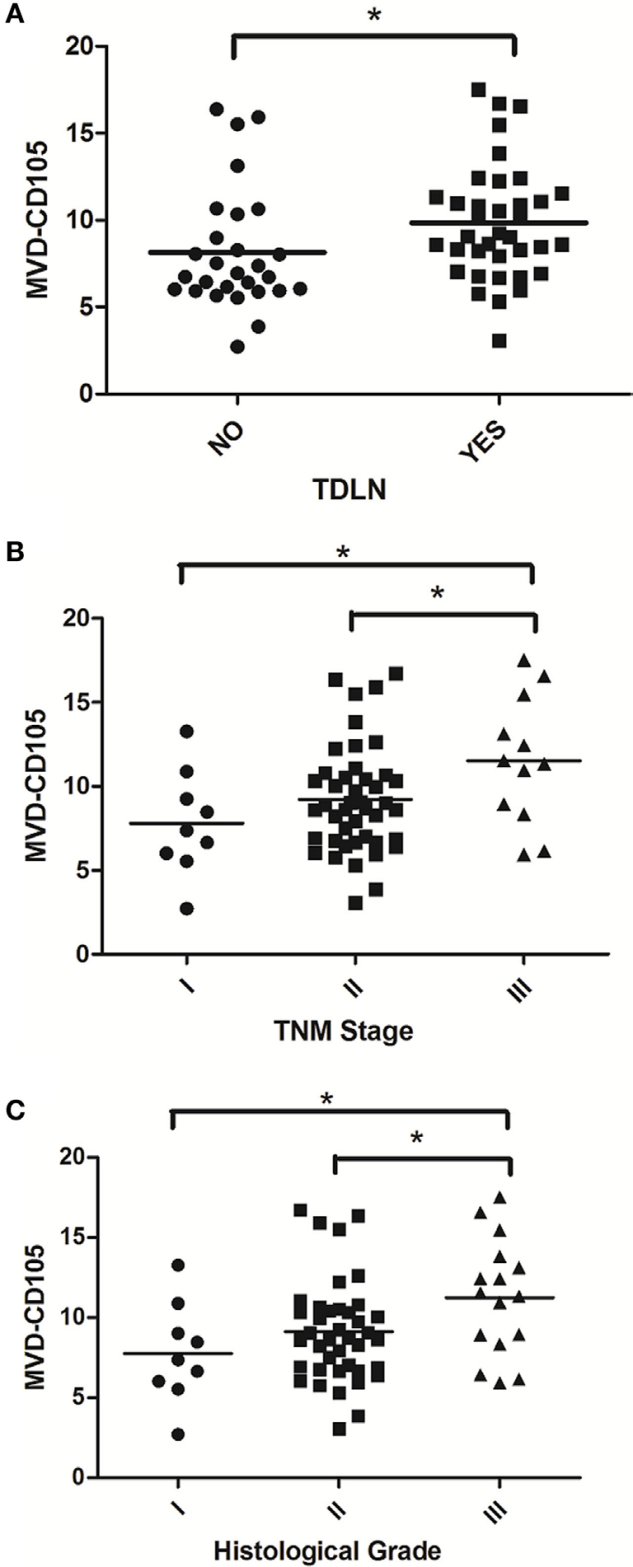
Scatter plot of microvessel density (MVD)-CD105 level associated with TDLNs metastasis, TNM stage and histological grade. The MVD-CD105 level in breast cancer patients with lymph nodes metastasis was significantly higher than those without lymph node metastasis **(A)**. The MVD-CD105 level was higher in stage III breast cancer patients than stage I or stage II patients **(B)**. The MVD-CD105 level was higher in histological grade III breast cancer patients than histological grade I or histological grade II patients **(C)**.

In order to evaluate the clinical significance of IDO expression in breast cancer, a univariate analysis was performed between the IDO or CD105 and clinicopathological features of the same patient, respectively. As shown in Figure 3, both IDO expression and MVD-CD105 level were associated with TNM stage, histological grade and TDLNs metastasis (*P* < 0.05). The positive rates of IDO expression in stage I, stage II, or stage III breast cancer were 22.22, 70.45, and 75.00%, respectively (*P* < 0.05), while the mean MVD levels were 8.27 ± 1.34, 9.52 ± 1.63, and 10.22 ± 2.23, respectively (*P* < 0.05). The positive rates of IDO expression in histological grade I, histological grade II, and histological grade III breast cancer were 22.00, 70.00, and 75.00%, respectively (*P* < 0.05), while the mean MVD levels were 8.26 ± 1.76, 9.41 ± 1.48, and 10.17 ± 1.86, respectively (*P* < 0.05) (Table 2). Similarly, the positive rate of IDO expression in breast cancer patients with lymph nodes metastasis was significantly higher than those without lymph node metastasis, which were 76.00 and 50.00%, respectively (*P* < 0.05), while the mean MVD levels were 9.87 ± 1.89 and 8.81 ± 2.19, respectively (*P* < 0.05). In contrast, there was no significant correlation between the IDO expression and other clinical and pathological indexes, such as age and expression of ER, PR, or Her2. We also did not find any correlation between IDO or MVD and the therapeutic approach of the breast cancer patients.

**Table 2:** The association between indoleamine 2,3-dioxygenase (IDO) expression, microvessel density (MVD)-CD105 level and clinicopathological features.

		IDO				MVD		
Clinicopathological features	*N* = 65	Negative	Positive	χ^2^	*P*	Mean ± SD	Value	*P*
**Age (years)**								
≤51	33	12	21	0.028	1.000	9.67 ± 2.51	*t* = 0.693	0.49
>51	32	11	21			9.21 ± 2.83		
**TDLNs metastasis**								
No	28	14	14	4.596	0.032	8.81 ± 2.19	*t* = 2.090	0.041
Yes	37	9	28			9.87 ± 1.89		
**TNM stage**								
I	9	7	2	9.309	0.025	8.27 ± 1.34	*F* = 2.309	0.034
II	44	13	31			9.52 ± 1.63		
III	12	3	9			10.22 ± 2.23		
**Histological grade**								
I	9	7	2	8.075	0.018	8.26 ± 1.76	*F* = 2.635	0.026
II	40	12	28			9.41 ± 1.48		
III	16	4	12			10.17 ± 1.86		
**ER-status**								
−	20	10	10	2.465	0.157	9.16 ± 2.84	*t* = 0.757	0.452
+	41	12	29			9.57 ± 1.41		
**PR-status**								
−	24	12	12	3.311	0.102	9.30 ± 2.57	*t* = 0.327	0.745
+	37	10	27			9.52 ± 2.56		
**Her-2-status**								
−	19	6	13	0.921	0.820	9.16 ± 2.13	*t* = 0.641	0.524
+	42	16	26			9.56 ± 2.31		

*For categorical variables, the χ^2^ value and P value were calculated by Chi-square tests; for continuous variables, values are given as mean ± SD and compared with independent t test*.

*TDLNs, tumor-draining lymph nodes; TNM, T category describes the primary tumor site; N category describes the regional lymph node involvement; M category describes the presence or otherwise of distant metastatic spread*.

### Prognostic Analysis

**Figure 4:**
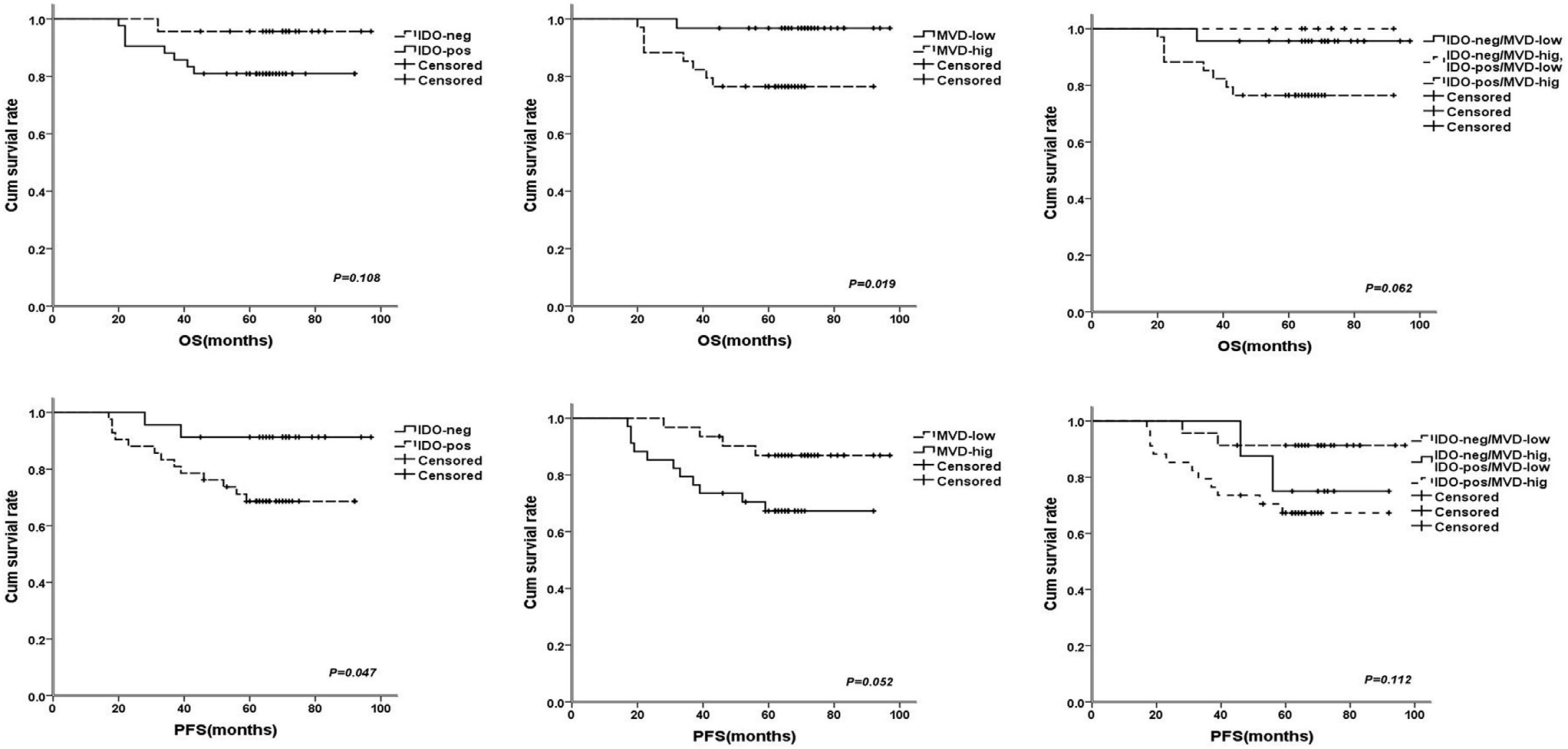
The survival curves for breast cancer patients after 68 months (range: 20–97 months) follow-up times, the patients with indoleamine 2,3-dioxygenase (IDO) expression or high microvessel density (MVD) level had poorer prognosis compared with no IDO expression [*P* = 0.108 for OS and *P* = 0.047 for progress-free survival (PFS)] and low MVD level (*P* = 0.019 for OS and *P* = 0.052 for PFS); the patients with IDO expression and high MVD level had a tendency with shorter overall survival (*P* = 0.062 for OS and *P* = 0.112 for PFS).

Univariate analysis revealed that TDLNs metastasis, an advanced TNM stage (III) and high histological grade (III) significantly predicted shorter 5-year survival rate of progress-free survival (PFS) and overall survival (OS) (Table 3). Concentrating on IDO and MVD, the patients with IDO expression or high MVD level had poorer prognosis compared with no IDO expression (*P* = 0.047 for PFS) and low MVD level (*P* = 0.019 for OS); the patients with IDO expression and high MVD level had a tendency with shorter OS when compared with non IDO expression, low MVD level or both (*P* = 0.062 for OS) (Figure 4).

**Table 3:** Univariate and multivariate analyses for progress-free survival (PFS) and overall survival (OS).

Univariate analyses for PFS and OS					
Parameter		5-year survival rate of PFS (%)	*P*	5-year survival rate of OS (%)	*P*
Age (years)	≤51 vs. >51	75.1 vs. 86.2	0.365	78.8 vs. 92.9	0.081
Tumor-draining lymph nodes (TDLNs) metastasis	No vs. Yes	95.5 vs. 69.3	0.001	100 vs. 75.7	0.006
TNM stage	I, II vs. III	92.8 vs. 36.5	0.000	95.7 vs. 55.9	0.000
Histological grade	I, II vs. III	89.8 vs. 38.4	0.000	93.4 vs. 45.5	0.000
Indoleamine 2,3-dioxygenase (IDO)	Negative vs. positive	91.5 vs. 74.0	0.047	94.3 vs. 79.9	0.108
Microvessel density (MVD)	Low vs. high	89.8 vs. 72.3	0.052	95.0 vs. 77.0	0.019
IDO and MVD	IDO-neg/MVD-low vs. IDO-neg/MVD-low, IDO-pos/MVD-high vs. IDO-pos/MVD-high	91.5 vs. 81.6 vs. 72.3	0.112	95.5 vs. 100 vs. 75.3	0.062
ER-status	Positive vs. negative	84.7 vs. 76.9	0.648	90.5 vs. 81.4	0.455
PR-status	Positive vs. negative	82.9 vs. 79.6	0.969	90.6 vs. 82.9	0.403
Her-2-status	Positive vs. negative	78.1 vs. 85.4	0.487	83.6 vs. 90.1	0.557

**Multivariate analyses for PFS and OS**					

**Parameter**		**HR (95%CI) for PFS**	***P***	**HR (95%CI) for OS**	***P***

TDLNs metastasis	No vs. Yes	0.138 (0.013–1.469)	0.101	0.000 (0.000–199.799)	0.954
TNM stage	I vs. II vs. III	0.126 (0.024–0.669)	0.015	0.102 (0.009–1.159)	0.066
Histological grade	I vs. II vs. III	0.840 (0.214–3.291)	0.802	0.733 (0.144–3.738)	0.709
IDO	Negative vs. positive	0.250 (0.020–3.098)	0.280	–	–
MVD	Low vs. high	3.442 (0.282–41.957)	0.333	0.411 (0.049–3.421)	0.411

In multivariate analysis, an advanced TNM stage (III) was significantly associated with shorter 5-year survival rate of PFS (HR: 0.126, 95% CI: 0.024–0.669, *p* = 0.015). Despite their effects on PFS and OS in univariate analysis, expression of IDO and level of MVD were not consistently associated with 5-year PFS and OS, suggesting a limited prognostic effect (Table 3).

### IDO in Breast Cancer Cell Lines

**Figure 5:**
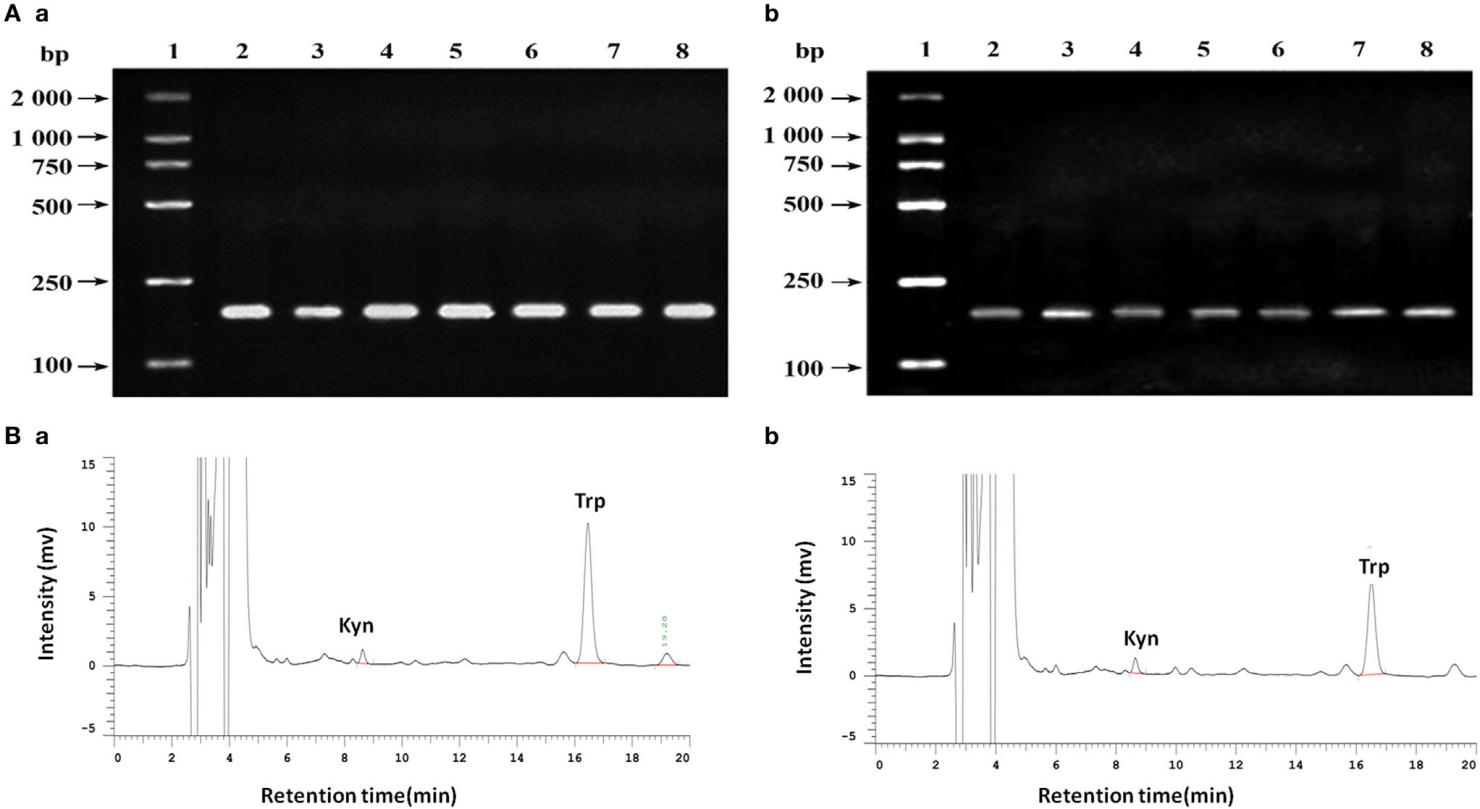
**(A)** Analysis of indoleamine 2,3-dioxygenase (IDO) expression in different cell lines by agarose gel electrophoresis. (a) β-actin; (b) IDO. 1. DNA marker DL2000; 2. MDA-MB-231; 3. MDA-MB-435S; 4. MDA-MB-453; 5. SK-Br-3; 6. ZR-75-1; 7. T47D; 8. MCF-7. **(B)** Analysis of tryptophan and kynurenine by high performance liquid chromatography using reversed phase C18 columns (5 µm, 4.6 mm × 150 mm, Kromasil). Retention times for kyn and try were 8.65 and 16.51 min, respectively. The concentration of tryptophan and kynurenine in MCF-7 cells the culture supernatant were 2.070 ± 0.604 mg/L and 0.207 ± 0.012 mg/L (b), while 4.707 ± 0.051 mg/L and 0.193 ± 0.006 mg/L in no cell culture supernatant (a).

In the retrospective study, we observed the correlation between IDO and MVD in breast cancer using tissue samples. In order to verify the phenomenon of IDO promoting angiogenesis, we contained the study *in vitro*. IDO mRNA expression were detected in all of the seven breast cancer cell lines, MDA-MB-231, MDA-MB-435S, MDA-MB-453, SK-Br-3, ZR-75-1, T47D, and MCF-7 (Figure 5A). The test of tryptophan and kynurenine were repeated three times by HPLC. The concentration of tryptophan and kynurenine in the culture supernatant of MCF-7 cells were 2.070 ± 0.604 mg/L and 0.207 ± 0.012 mg/L (Figure 5Bb), while 4.707 ± 0.051 mg/L and 0.193 ± 0.006 mg/L in no cell culture supernatant (Figure 5Ba). The ratio of kyn/trp in the culture supernatant of MCF-7 cells were nearly doubled (99.810 ± 4.115 vs. 52.154 ± 1.132) compared with the control, indicating that IDO expressed by MCF-7 cells possessed functional activity and could metabolize tryptophan (Figure 5B).

### IDO on HUVEC Cell Proliferation and Expression of CD105

**Figure 6:**
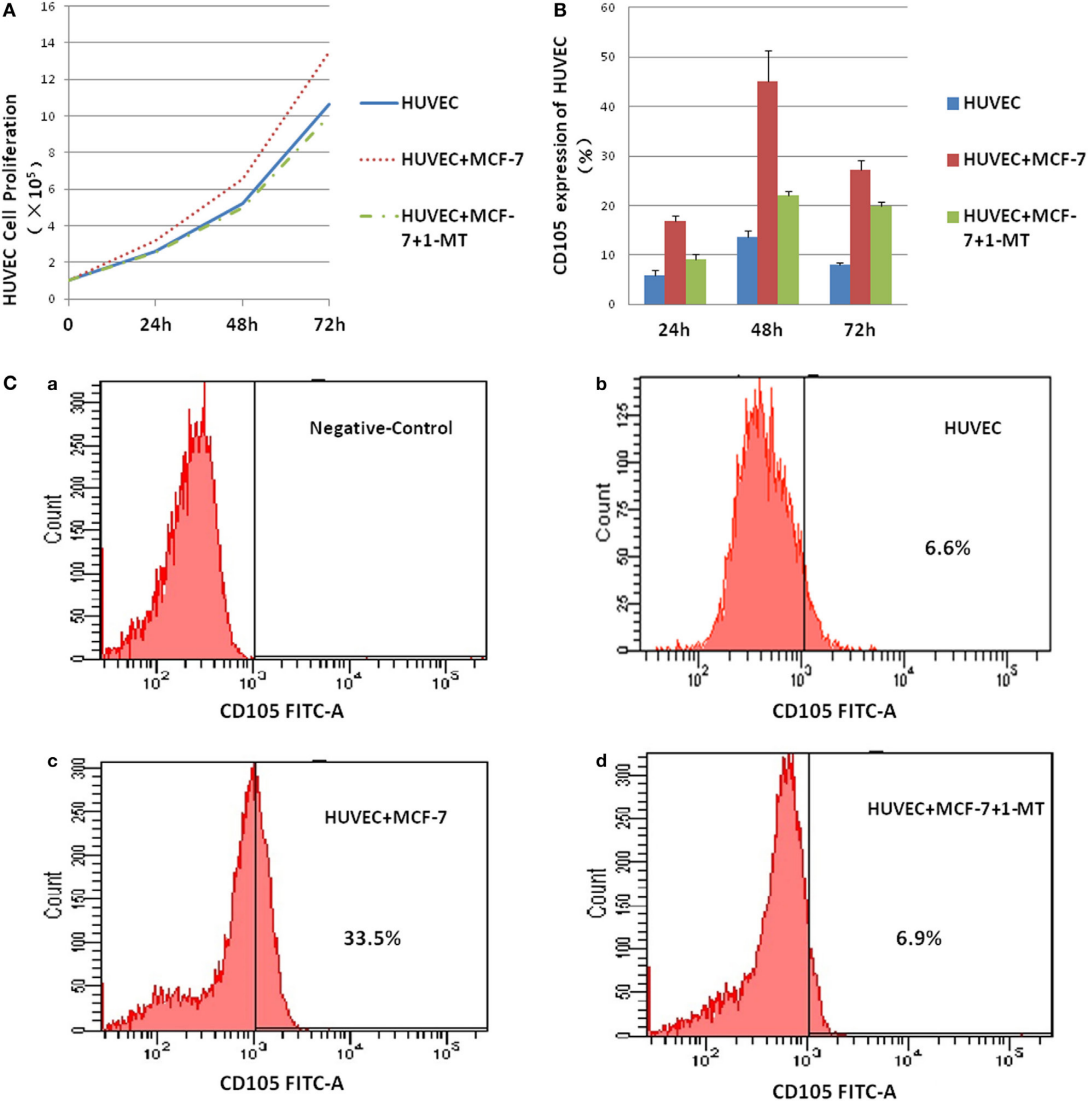
Indoleamine 2,3-dioxygenase (IDO) on human umbilical vein endothelial cells (HUVEC) activity. Co-culture of MCF-7 and HUVEC was performed in the presence or absence of the IDO inhibitor 1-methyl-d-trytophan (1-MT). **(A)** HUVEC proliferation was measured using the Cell Counting, IDO promoted HUVEC cell proliferation significantly compared with control after co-culture for 3 days. **(B)** CD105 expression by HUVEC in the control [**(C)** (a)], HUVEC groups [**(C)** (b)], HUVEC + MCF-7 groups [**(C)** (c)], and HUVEC + MCF-7 + 1-MT groups [**(C)** (d)] were detected by flow cytometry. CD105, which was upregulated expression in HUVEC + MCF-7 group compared with control group, was downregulate expressed after adding inhibitor 1-methyl-d-trytophan.

To test the role of IDO on HUVEC, co-culture system was performed in the presence or absence of the IDO inhibitor 1-methyl-d-trytophan (1-MT). HUVEC proliferation was measured using Cell Counting. IDO promoted HUVEC cell proliferation significantly compared with control group after co-culture for 72 h (Figure 6A). CD105 expression by HUVEC in the control, HUVEC + MCF-7, and HUVEC + MCF-7 + 1-MT groups was detected by flow cytometry (Figure 6C). CD105 expression, which was upregulated in HUVEC + MCF-7 group compared with control group, was downregulated expressed after adding 1-MT (Figure 6B).

## Discussion

### IDO and Breast Cancer

As one of several immune checkpoints involved in tumor immune escape, IDO has emerged as a key target in cancer therapy because of immunoregulatory roles associated with tryptophan metabolism. Currently, inhibitors of the IDO pathways are rapidly advancing in the clinical (19). The preliminary efficacy of a study of Epacadostat (a potent and selective oral inhibitor of IDO 1) plus pembrolizumab in TNBC is only 10%, while the efficacy is 35% in non-small-cell lung cancer (NSCLC) (20). Therefore, it is very important to research the network of immunosuppressive mechanisms in the different tumor microenvironment, helping to identify the most effective way to target IDO for immunotherapy.

In our study, the IDO expression was correlated with initial TNM stage, histological grade and TDLNs metastasis, which was mainly consistent with previous studies. But in Soliman et al.’s study (21), IDO expression was higher in smaller, node-negative breast cancer compared to more advanced stages and was higher in ER+ tumors compared to ER− tumors. In our study, we did not find a correlation between IDO expression and ER, PR, or Her-2 status of breast cancer patients. Jacquemier et al. (22) evaluated IDO expression in a large cohort of patients with triple-negative breast cancer (TNBC) and examined the clinicopathologic characteristics of the subset of the IDO1-expressing TNBC subset. They reported that high IDO expression was associated with morphological medullary features and had an independent favorable prognostic value in basal-like breast carcinoma. Concentrating on the prognostic effects of IDO and MVD, the survival curves showed that the patients with IDO expression and high MVD level had a tendency with shortest OS time when compared with non IDO expression, low MVD level, or both in univariate analysis. Despite their affection on PFS and OS in univariate analysis, expression of IDO and level of MVD were not consistently associated with 5-year PFS and OS in multivariate analysis, suggesting a limited prognostic effect in our study. Further studies with longer follow-up and larger sample size are needed to establish whether IDO could be a valuable diagnostic and prognostic biomarker.

### Angiogenesis and Breast Cancer

Angiogenesis is a complex biological process that plays a crucial role in tumor development and metastasis. There are many biomarkers to label MVD to evaluate angiogenesis in tumor microenvironment, such as CD31 and CD105 (22), but it is still unclear which is a better marker for disease progression and prognosis.

Deliu et al. (23) found that the more blood vessels were marked with CD34 than CD31 or CD105. In addition, CD31 expression was associated with CD105 expression in colorectal cancer. One limitation of Deliu et al.’s study is that the relationship between MVD and prognosis was not examined. Jyothsna et al. (24) reported that MVD measured by CD31 may be used as indicators for disease progression of oral squamous cell carcinomas. Interestingly, another study published by Dales et al. (25) demonstrated that CD105 was associated with greater risk of metastasis and worse outcome in breast cancer patients. In our study, we stained CD31 and CD105 as markers of MVD in breast cancer tissues, showing that MVD-CD105 level was associated with initial TNM stage, histological grade, and TDLNs metastasis in breast cancer, while MVD using CD31 was not associated with clinicopathological features (not shown). Meanwhile we found that the IDO expression was correlated with the level of MVD assessed by CD105, but not CD31 staining. Therefore, it is speculated that IDO may promote the progression of breast cancer though promoting angiogenesis excepting immune escape.

The understanding of the molecular mechanisms of angiogenesis has increased drastically in the past decade. To date, two antiangiogenic monoclonal antibodies, bevacizumab and ramucirumab, are approved for the treatment of patients with advanced or metastatic NSCLC in combination with chemotherapy (26). However in breast cancer, the value of bevacizumab has been debated for several years (27). Recent studies have identified new targets and strategies that may be helpful for improving the efficacy of antiangiogenic treatments. There may be other mechanisms of angiogenesis in breast cancer that can be targeted. As with Li et al.’s research (13), IDO may have the effect of inducing angiogenesis through the depletion of tryptophan, promoting the engraftment of skin substitutes.

### IDO and MVD

Indoleamine 2,3-dioxygenase activity in tumors is deemed as an attractive therapeutic target to recover immunity against cancer, but the data on IDO non-immune functions are lacking. Smith et al. (28) reported that IDO may affect inflammation, vascularization, and immune escape and therefore may be associated with primary and metastatic tumor growth. In their research, noninvasive micro-computed tomographic scans were conducted to assess the impact of IDO1 loss on overt lung tumors. The authors found that in IDO1-nullizygous mice the density of pulmonary blood vessels was reduced.

De Palma et al. reported that the tumor microenvironment may promote tumor angiogenesis (12). Few studies have examined the relationship between IDO expression and angiogenesis. Nonaka et al. (14) indicated that IDO expression was not associated with cancer cell growth and invasion *in vitro*, but IDO expression did promote tumor growth and peritoneal dissemination *in vivo* through inhibiting NK cell accumulation and promoting tumor angiogenesis. In our study, we analyzed the correlation between IDO and MVD-CD105 in breast cancer tissue and find that both IDO expression and MVD-CD105 level were associated with initial TNM stage, histological grade, and TDLNs metastasis. We found a positive correlation between IDO and MVD-CD105, but not MVD-CD31. But Soliman et al. (21) revealed an inverse relationship between CD31 and IDO expression in breast cancer, suggesting that IDO was lower in those with greater neoangiogenesis. In their analysis, it appeared that tumors with higher IDO levels had higher expression of p-caspase 9, EGFR, NOS2, and COX2.

*In vitro*, we also verified that the treatment with supernatants of MCF-7 cells can cause HUVEC to proliferate and express CD105. We also found that the ability of IDO to upregulate the expression of CD105 could be suppressed by 1-MT. Similar to our finding, Su et al. found that overexpression of IDO could promote the attachment of 2LL cells, the ability of migration, invasion, and VM formation.

Indoleamine 2,3-dioxygenase expression has been reported in a range of cancers (29), which is characterized in tumor cells and also has been described in a variety of immune cells. Although many researchers have concentrated on the mechanism of IDO promoting tumor progression, it remains unclear whether and how IDO participates in promoting cancer angiogenesis. Marti et al.’s study indicated that VEGF increased the expression and activity of IDO in DCs (30). Thus, we infer that IDO may play an important role in the process of VEGF promoting angiogenesis. But another study (31) found that long non-coding RNA MALAT1 on mesenchymal stem cells not only induced IDO expression but also promoted tube formation of HUVEC mediated by VEGF.

In the present study, we found a positive correlation between IDO and MVD-CD105 in breast cancer tissue, and both IDO expression and MVD-CD105 level were associated with initial TNM stage, histological grade and TDLNs metastasis. Meanwhile, *in vitro*, we found that IDO can promote the HUVEC cell growth and the expression of CD105. However, it was a retrospective study of relatively small sample. In addition, we will further validate these findings with animal models and explore the mechanism of IDO promoting angiogenesis. Targeting IDO may lead to a novel molecular or gene therapy targeting angiogenesis inhibition in the future.

## Ethics Statement

Ethics approval and consent to participate: this research project was approved by the Ethics Committee of Tianjin Cancer Institute and Hospital. Written consents were obtained from each patient. Consent for publication: written consents were obtained from each patient for publishing their pathological images as represent figures.

## Author Contributions

LW, JL, and XR analyzed and interpreted the patient data. LW and SZ performed the immunohistochemistry and histological examination of the cancer tissues. LW and ML performed the cell experiments and flow cytometry. LW was a major contributor in writing the manuscript. FL and FW was analyzed and interpreted and revised the manuscript. All authors read and approved the final manuscript.

## Conflict of Interest Statement

The authors declare that the research was conducted in the absence of any commercial or financial relationships that could be construed as a potential conflict of interest.

## Supplementary Material

Figure S1 Indoleamine 2,3-dioxygenase protein expression was not detected in normal tissues by immunohistochemistry. Original magnification, ×200.
