# Supplementary figures and images for: High Indoleamine 2,3-Dioxygenase Is Correlated With Microvessel Density and Worse Prognosis in Breast Cancer
Source: Front Immunol. 2018 Apr 17;9:724. doi: 10.3389/fimmu.2018.00724 (PMC5913323; doi:10.3389/fimmu.2018.00724)

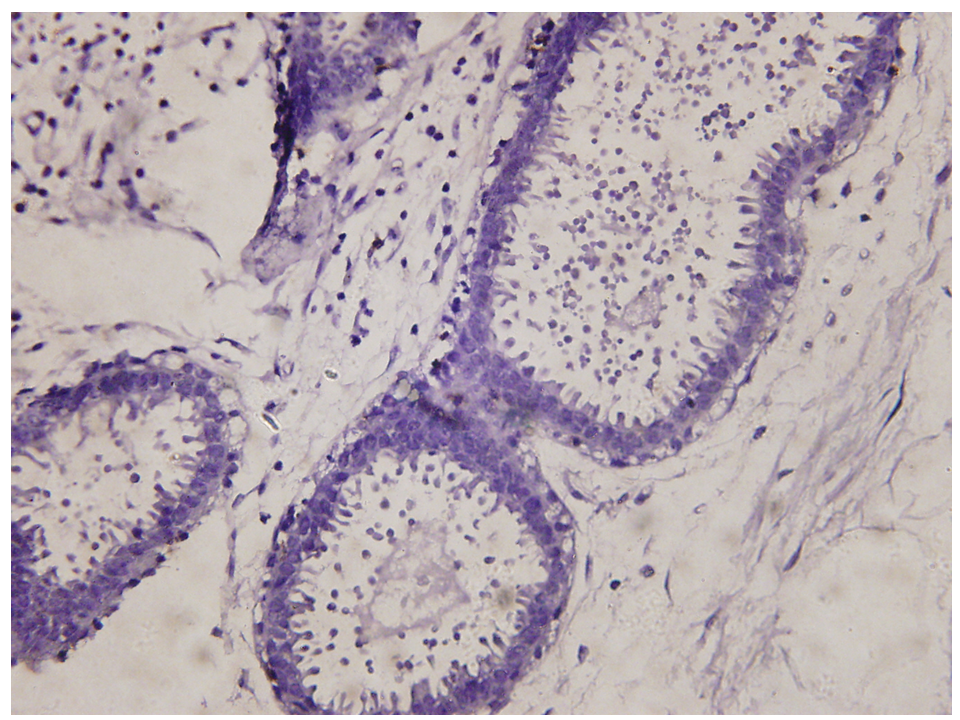

Supplement: Figure S1 — Indoleamine 2,3-dioxygenase protein expression was not detected in normal tissues by immunohistochemistry. Original magnification, ×200. [file image_1.tif]
